# Association of *TOP2A* and *ADH1B* with lipid levels and prognosis in patients with lung adenocarcinoma and squamous cell carcinoma

**DOI:** 10.1111/crj.13717

**Published:** 2023-11-20

**Authors:** Dongdong Yin, Yinci Zhang, Hui Li, Longqiang Cheng

**Affiliations:** ^1^ First Affiliated Hospital (Huainan First People's Hospital) Anhui University of Science and Technology Huainan China

**Keywords:** alcohol dehydrogenase 1B, lung adenocarcinoma, lung squamous cell carcinoma, topoisomerase II alpha, total cholesterol, triglyceride

## Abstract

**Background:**

Although lung adenocarcinoma (LUAD) and lung squamous cell carcinoma (LUSC) have different pathological and clinical features, they may share common driver genes. It was found that lipid levels can be used for early diagnosis of NSCLC; however, the relationship between driver genes and genes regulating lipid metabolism and their relationship with patient prognosis needs further investigation.

**Methods:**

Genes whose expression was up‐ or down‐regulated in both LUAD and LUSC were identified using the GEO database. Online tools like GEPIA 2, PrognoScan, UALCAN, and TIMER2.0 were used to investigate the association of these gene expressions with the patient's prognosis and lipid regulatory genes. The association between clinical lipid levels and the risk of LUAD and LUSC was analyzed by using a multiple logistic regression model.

**Results:**

Topoisomerase II alpha (*TOP2A*) and alcohol dehydrogenase 1B (*ADH1B*) were identified as the only genes up‐ and down‐regulated in both LUAD and LUSC. *TOP2A* and *ADH1B* expression levels significantly correlated with the patient's gender, age, individual cancer stage, histological subtype, nodal metastasis status, and TP53 mutation status. Additionally, only LUAD patients with higher *TOP2A* or lower *ADH1B* expressions displayed poor overall and relapse‐free survival rates. Moreover, *TOP2A* levels exhibited a negative correlation with adipose triglyceride lipase (*ATGL*) and ATP‐binding cassette transporter A1 (*ABCA1*) in both LUAD and LUSC. However, *ADH1B* showed inverse associations with the above‐mentioned genes when compared to *TOP2A* expressions in both LUAD and LUSC. Furthermore, elevated triglyceride (OR = 1.59; 95% CI = 1.01 to 2.49; *P* < 0.05) and total cholesterol (OR = 2.45; 95% CI = 1.08 to 5.57; *P* < 0.05) levels might increase the risk of LUAD.

**Conclusions:**

*TOP2A* and *ADH1B* can be used as diagnostic markers for LUAD and LUSC, but only as independent prognostic factors for LUAD, and may be involved in lipid metabolism in LUAD patients but not in LUSC. Thus, combining genetic diagnostics with lipid panel tests might be an effective method for an early diagnosis and improved prognosis of LUAD.

## Graphical abstract

*TOP2A* and *ADH1B* were identified as the only genes up‐ and down‐regulated in both LUAD and LUSC. *TOP2A* and *ADH1B* can be used as diagnostic markers for LUAD and LUSC, but only as independent prognostic factors for LUAD, and may be involved in lipid metabolism in LUAD patients but not in LUSC.

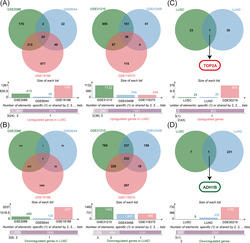

## 1 INTRODUCTION

Lung cancer is a leading cause of death worldwide, accounting for 11.6% of the total affected population in 2018.^1^ The incidence and mortality rates of lung cancer in China were reported to be as high as 17.9% and 23.8% in 2021, thereby making lung cancer a major public health and social burden.^2^ The lack of early symptoms and increased advanced‐stage cases at diagnosis could be the main reasons that hamper the timely diagnosis and treatment of lung cancer. Non‐small cell lung cancer (NSCLC) is the most common type of lung cancer, accounting for 85% of all cases,^3^ with a poor prognosis^4^ and a 5‐year survival rate of <15%.^5^ Therefore, an early diagnosis of NSCLC is crucial for improving its prognosis. NSCLC can be further subdivided into two most common subtypes: lung adenocarcinoma (LUAD) and lung squamous cell carcinoma (LUSC). They both display different pathological and clinical features, metabolic regulation, and gene expression profiles. Subsequently, the researchers often overlook differences in key genes common to both subtypes, like metabolic regulation genes related to prognosis. Hence, a precise diagnosis and early treatment implementation might improve the patient's prognosis.

The topoisomerase II alpha (*TOP2A*) gene helps in chromosome condensation and segregation, along with DNA transcription and replication, by encoding the DNA topoisomerase II alpha enzyme.^6^ The alcohol dehydrogenase 1B (*ADH1B*) gene encodes a protein that is a member of the ADH family and helps in the regulation of alcohol metabolism, human behavior, liver function, and human evolution.^7^, ^8^ Recent evidence has shown that *TOP2A* and *ADH1B* genes are involved in the progression of several tumors. For example, a study confirmed that enhanced *TOP2A* gene expression might lead to poor prognosis in hepatocellular carcinoma patients.^9^ Moreover, *TOP2A*, as a downstream target of microRNA‐599, enhances the tumorigenic phenotype of bladder cancer cells.^10^ In another study, *TOP2A* promoted tumorigenesis in high‐grade plasmacytoid ovarian cancer via the TGF‐β/Smad pathway.^11^ A recent study has found that the *ADH1B* gene plays a crucial role in immune regulation and therapeutic response in ovarian cancer.^12^ Additionally, *ADH1B* gene polymorphism also increases the risk of several tumors, including hepatocellular carcinoma,^13^ esophageal cancer,^14^ and nasal cavity cancer,^15^ by regulating the acetaldehyde metabolism and alcohol intake propensity. In our study, *TOP2A* and *ADH1B* were identified as up‐and‐down‐regulated common genes of LUAD and LUSC, respectively. However, none of the studies have analyzed the combination of *TOP2A* and *ADH1B* as molecular markers for the diagnosis and prognosis of LUAD and LUSC to date.

Since lipid metabolism is an important physiological process, its abnormalities are associated with several pathophysiological changes in multiple diseases, such as hyperlipoproteinemia^16^ and pulmonary fibrosis.^17^ Recently, Wang et al.^18^ revealed abnormal lipid metabolism in lung cancer patients by utilizing scRNA‐seq and lipidomics. Thus, the detection of abnormal lipid metabolism can be duly used for the early detection of lung cancer or mass screening of high‐risk groups for cancer prevention. However, it is not known whether *TOP2A* and *ADH1B* can also affect lipid metabolism in lung cancer patients with different subtypes.

In our study, we first analyzed *TOP2A* and *ADH1B* expressions in LUAD and LUSC and their associations with the patient's prognosis. Then, combined with the common clinical lipid level assay indexes, we identified the lipid metabolism regulatory genes by reviewing the literature and analyzing their association with the pivotal genes. Furthermore, lipid panel tests were conducted from LUAD and LUSC patients' serums to investigate the association of altered lipid metabolism with the risk of LUAD or LUSC. This study aims to provide a clinical basis for the early and specific diagnosis of NSCLC and an improvement in its prognosis by analyzing the potential relationship between *TOP2A* and *ADH1B* genes and lipid metabolism as well as prognosis in NSCLC patients.

## 2 MATERIALS AND METHODS

### 2.1 Identification of intersecting LUAD and LUSC genes

First, GSE2088, GSE6044, and GSE19188, as well as GSE31210, GSE43458, and GSE118370, were selected as representative datasets for LUSC and LUAD from the Gene Expression Omnibus (GEO) database (http://www.ncbi.nlm.nih.gov/geo/),^19^ an international repository of functional genomics datasets. GSE2088 contained 48 LUSC and 30 normal tissues, GSE6044 included 24 LUSC and 5 normal tissues, and GSE19188 included 27 LUSC and 65 normal tissues. Similarly, GSE31210 contained 226 LUAD and 20 normal tissues, GSE43458 encompassed 110 LUAD and 30 normal tissues, and GSE118370 included 6 LUAD and 6 normal tissues. The inclusion criteria were as follows: (1) patients with LUSC and LUAD tissue sample datasets, (2) those having datasets with complete genetic testing technique information, and datasets (3) having normal controls. Thus, with these criteria and using principal component analysis as quality control, the above‐mentioned three datasets, each for LUSC and LUAD, were downloaded from the repository after three reviews by four researchers.

Then, differentially expressed genes (DEGs) between lung cancer and normal tissue samples were retrieved by an online analysis tool, GEO2R (https://www.ncbi.nlm.nih.gov/geo/geo2r/) at *p* < 0.05. Finally, the common DEGs between LUAD and LUSC representative datasets were obtained by another online analysis tool, jvenn (https://jvenn.toulouse.inrae.fr/app/example.html).

### 2.2 Analysis of intersecting LUAD and LUSC gene expression

First, the differences in *TOP2A* and *ADH1B* expressions, the LUAD and LUSC crossover genes, were analyzed in tumor and normal tissues, respectively, using online tools like GEPIA 2 (http://gepia2.cancer-pku.cn/#index) and TIMER2.0 (http://timer.comp-genomics.org/). Second, in the GEPIA 2 database, 483 LUAD and corresponding 347 normal tissue samples, as well as 486 LUSC and corresponding 338 normal tissue samples, were included for comparing *TOP2A* and *ADH1B* expressions in LUAD and LUSC and normal tissue samples. Lastly, in the TIMER2.0 database, 515 LUAD and corresponding 59 normal tissue samples, as well as 501 LUSC and corresponding 51 normal tissue samples, were selected for comparing *TOP2A* and *ADH1B* expressions in LUAD and LUSC and normal tissue samples. Additionally, the ranking of *TOP2A* and *ADH1B* expressions in LUAD and LUSC were analyzed by UALCAN, an online tool (https://ualcan.path.uab.edu/index.html), respectively. Finally, the correlation of *TOP2A* and *ADH1B* expression levels in LUAD and LUSC with the patient's gender, age, individual cancer stage, histological subtype, nodal metastasis status, and TP53 mutation status was analyzed using the UALCAN online tool, respectively.

### 2.3 Survival analysis of intersecting LUAD and LUSC genes

First, the GEPIA 2 online tool was used to analyze the effects of *TOP2A* and *ADH1B* genes on the overall survival (OS) and disease‐free survival (DFS) in LUAD and LUSC patients, respectively. Subsequently, based on the PrognoScan online database (dna00.bio.kyutech.ac.jp/PrognoScan/index.html), the GSE31210 dataset and 201292_at probe were selected for evaluating the effects of *TOP2A* on OS and relapse‐free survival (RFS) in LUAD patients, while GSE4573 dataset and 201292_at probe were selected to analyze the effect of *TOP2A* on OS in LUSC patients. Similarly, the GSE31210 dataset and 209612_s_at probe were selected to evaluate the effect of *ADH1B* on OS and RFS in LUAD patients, whereas the GSE4573 dataset and 209612_s_at probe were utilized to analyze the effect of *ADH1B* on OS in LUSC patients.

### 2.4 Correlation analysis of intersecting LUAD and LUSC genes with lipid metabolism regulatory genes

The correlations between the common driver genes of LUAD and LUSC and the clinical lipid metabolism regulatory genes adipose triglyceride lipase (*ATGL*), bacillus subtilis protease/kexin type 9 (*PCSK9*), ATP‐binding cassette transporter protein A1 (*ABCA1*), apolipoprotein a1 (*APOa1*), apolipoprotein b (*APOb*), and *LPa* in LUAD and LUSC were analyzed using Pearson's correlation coefficients in the GEPIA 2 online tool. Thus, −1 < R < 0 indicated a negative correlation, and 0 < R < 1 indicated a positive correlation; the larger the absolute value of R, the stronger the correlation was between the two genes.

### 2.5 Patient enrollment and clinical data collection

The clinical data of 76 NSCLC patients who attended the First Affiliated Hospital (Huainan First People's Hospital) of Anhui University of Science and Technology from January 2021 to December 2022 were retrospectively analyzed. All lung cancer patients were definitively diagnosed by histopathological or imaging confirmation, including 47 and 29 cases of LUAD and LUSC, respectively. The lung cancer staging was based on the International Association for the Study of Lung Cancer (IASLC) staging criteria, 8th edition, 2017.^20^ The general and clinical characteristics of the 76 NSCLC patients are shown in Table 1. Moreover, 61 non‐lung cancer patients examined during the same period were selected as controls. The age and gender of the two groups were similar (*P* > 0.05) and comparable (Table 2). The exclusion criteria were as follows: (1) patient's age <20 years or >95 years; (2) pregnant or breastfeeding women; (3) patients with previous lung cancer treatment; (4) those with external usage of lipid‐lowering drugs; (5) patients having severe chronic diseases such as chronic kidney disease or hyperparathyroidism; (6) those with other malignancies or immunodeficiency syndromes; (7) patients receiving systemic steroid therapy; (8) those already treated with surgery, interventional procedures, radiotherapy, chemotherapy, targeted drugs, and so forth at the time of enrolment; and patients (9) with missing clinical data. This study was approved by the Human Research Ethics Committee of the First Affiliated Hospital (Huainan First People's Hospital) of Anhui University of Science and Technology, China (No. EC‐20221028‐1013), and individual consent for this retrospective analysis was waived.

**TABLE 1:** General and clinical characteristics of NSCLC patients.

	*n*	%
Gender		
Male	49	64.5
Female	27	35.5
Age (mean ± SD)	63.00 ± 1.241	
Tumor site		
Right lung	41	53.9
Left lung	35	46.1
Histology type		
LUAD	47	61.8
LUSC	29	38.2
TNM staging		
1	45	59.2
2–4	31	40.8
Primary organ		
Y	74	97.4
N	2	2.6
Lymph node metastasis		
N0	55	72.4
N1–3	21	27.6
Distal organ metastasis		
M0	66	86.8
M1	10	13.2
Clinical Staging		
Stage I	51	67.1
Stages II–IV	25	32.9

Abbreviations: LUAD, lung adenocarcinoma; LUSC, lung squamous cell carcinoma; NSCLC, non‐small cell lung cancer.

**TABLE 2:** Relationship of blood lipid levels and NSCLC.

	Patients in the control group	NSCLC patients	*P* value
Gender^a^			0.379
Male	34 (55.7%)	49 (64.5%)	
Female	27 (44.3%)	27 (35.5%)	
Age (mean ± SD)^b^	60.28 ± 1.04	63.00 ± 1.24	0.105
TC^b^	1.12 ± 0.05	1.46 ± 0.10	0.007
TG^b^	4.15 ± 0.08	4.40 ± 0.14	0.126
HDL^b^	1.16 ± 0.04	1.08 ± 0.04	0.205
LDL^b^	2.68 ± 0.08	2.67 ± 0.11	0.959
APOa^b^	1.25 ± 0.02	1.22 ± 0.03	0.493
APOb^b^	0.77 ± 0.02	0.81 ± 0.03	0.237
Lp(a)^c^	156.50 (54.75, 440.0)	136.00 (73.00, 318.00)	0.149

Abbreviations: APOa, apolipoprotein a; APOb, apolipoprotein b; HDL, high‐density lipoprotein; LDL, low‐density lipoprotein; Lp(a), lipoprotein(a); NSCLC, non‐small cell lung cancer; TC, total cholesterol; TG, triglyceride.

^a^ Chi‐square tests.

^b^ An independent sample *t* test.

^c^ Nonparametric tests.

### 2.6 Statistical analysis

Statistical analysis was performed using SPSS 19.0 software (IBM, Armonk, NY, United States). A multiple logistic regression model was used to explore the association of TG and TC levels with the risk of developing LUAD and LUSC. All values pertaining to *P* < 0.05 indicated statistical significance.

## 3 RESULTS

### 3.1 Identification of *TOP2A* and *ADH1B* as intersecting genes of LUAD and LUSC based on multiple datasets

**FIGURE 1:**
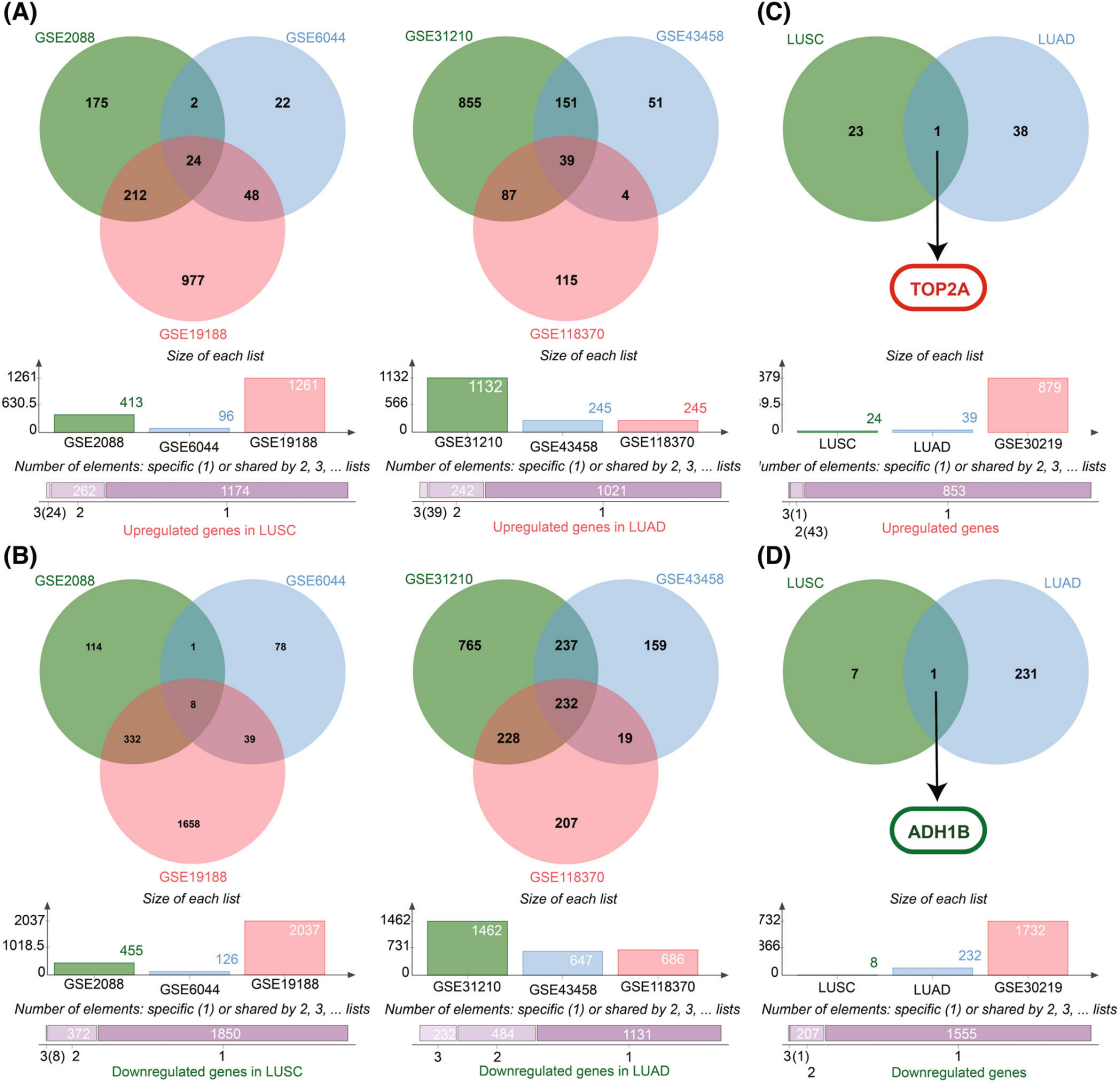
Identification of *TOP2A* and *ADH1B* as intersecting LUAD and LUSC genes, based on multiple datasets by the jvenn online analysis tool. DEGs were selected based on the criteria *P*adj < 0.05, |logFC| ≥ 1.0. *TOP2A*, topoisomerase II alpha; *ADH1B*, alcohol dehydrogenase 1B; DEGs, differentially expressed genes; LUAD, lung adenocarcinoma; LUSC, lung squamous cell carcinoma.

In LUSC, 24 upregulated and 8 downregulated intersection DEGs were obtained from the GSE2088, GSE6044, and GSE19188 datasets by the jvenn online analysis tool (Figure 1A). Moreover, in LUAD, 39 upregulated and 232 downregulated intersection DEGs were obtained from the GSE31210, GSE43458, and GSE118370 datasets by the jvenn online analysis tool (Figure 1B). Furthermore, 24 and 39 upregulated intersection DEGs of LUSC and LUAD were used to obtain one upregulated intersection gene, *TOP2A* (Figure 1C). Similarly, 8 and 232 down‐regulated intersection DEGs of LUSC and LUAD were utilized to obtain 1 down‐regulated intersection gene, *ADH1B* (Figure 1D).

### 3.2 Expression analysis of TOP2A and ADH1B genes in LUAD and LUSC based on multiple databases

**FIGURE 2:**
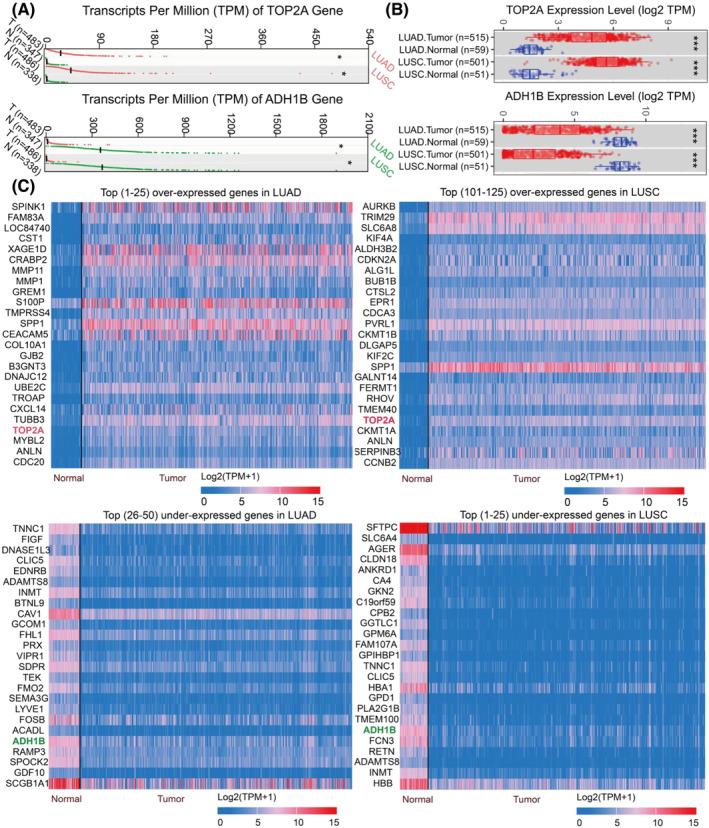
Evaluation of *TOP2A* and *ADH1B* expressions in LUAD and LUSC was investigated according to several databases. (A) *TOP2A* and *ADH1B* expressions in LUAD and LUSC were analyzed by the GEPIA 2 database. (B) *TOP2A* and *ADH1B* expressions in LUAD and LUSC were examined by the TIMER2.0 database. (C) Expressions of *TOP2A* and *ADH1B* in LUAD and LUSC were studied by the UALCAN database. *P < 0.05, ^***^ *P* < 0.001. *TOP2A*, topoisomerase II alpha; *ADH1B*, alcohol dehydrogenase 1B; LUAD, lung adenocarcinoma; LUSC, lung squamous cell carcinoma; N, Normal; T, tumor.

As shown in Figure 2A,B, the *TOP2A* gene was highly expressed in LUAD and LUSC tissue samples when compared to normal tissue samples, whereas decreased *ADH1B* expression was observed in LUAD and LUSC tissue samples. Additionally, *TOP2A* was determined as a TOP 25 and TOP 101–125 overexpressed gene in LUAD and LUSC cases, whereas ADH1B was a TOP 26–50 and TOP 1–25 low‐expressed gene in LUAD and LUSC cases, in the UALCAN database, respectively (Figure 2C). Thus, this suggested that *TOP2A* was highly expressed in LUAD and LUSC patients, while ADH1B displayed inadequate expression in LUAD and LUSC cases.

### 3.3 Analysis of clinical factors affecting *TOP2A* and *ADH1B* expressions in LUAD and LUSC

**FIGURE 3:**
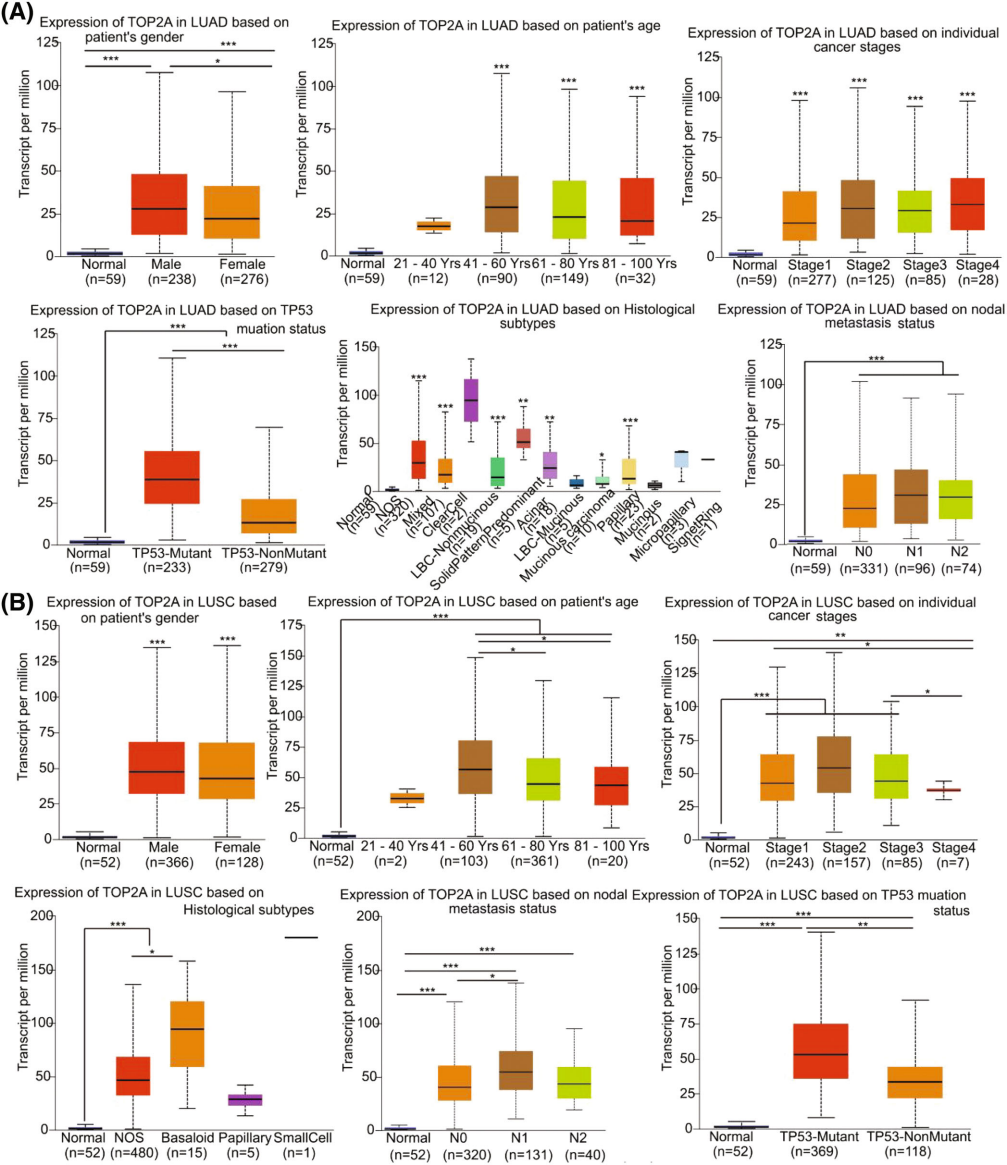
An analysis of the clinical factors affecting *TOP2A* expressions in LUAD and LUSC by the UALCAN database. (A) an analysis of the clinical factors affecting *TOP2A* expression in LUAD. (B) An analysis of the clinical factors affecting *TOP2A* expression in LUSC. *P < 0.05, ^**^ *P* < 0.01, ^***^ *P* < 0.001. *TOP2A*, topoisomerase II alpha; LBC‐mucinous, lung bronchioloalveolar carcinoma; LBC‐nonmucinous, lung bronchioloalveolar carcinoma non‐mucinous; LUAD, lung adenocarcinoma; LUSC, lung squamous cell carcinoma; Yrs, years; NOS, lung adenocarcinoma‐not otherwise specified; mixed, lung adenocarcinoma mixed subtype; N0, no regional lymph node metastasis; N1, metastases in 1 to 3 axillary lymph nodes; N2, metastases in 4 to 9 axillary lymph nodes.

**FIGURE 4:**
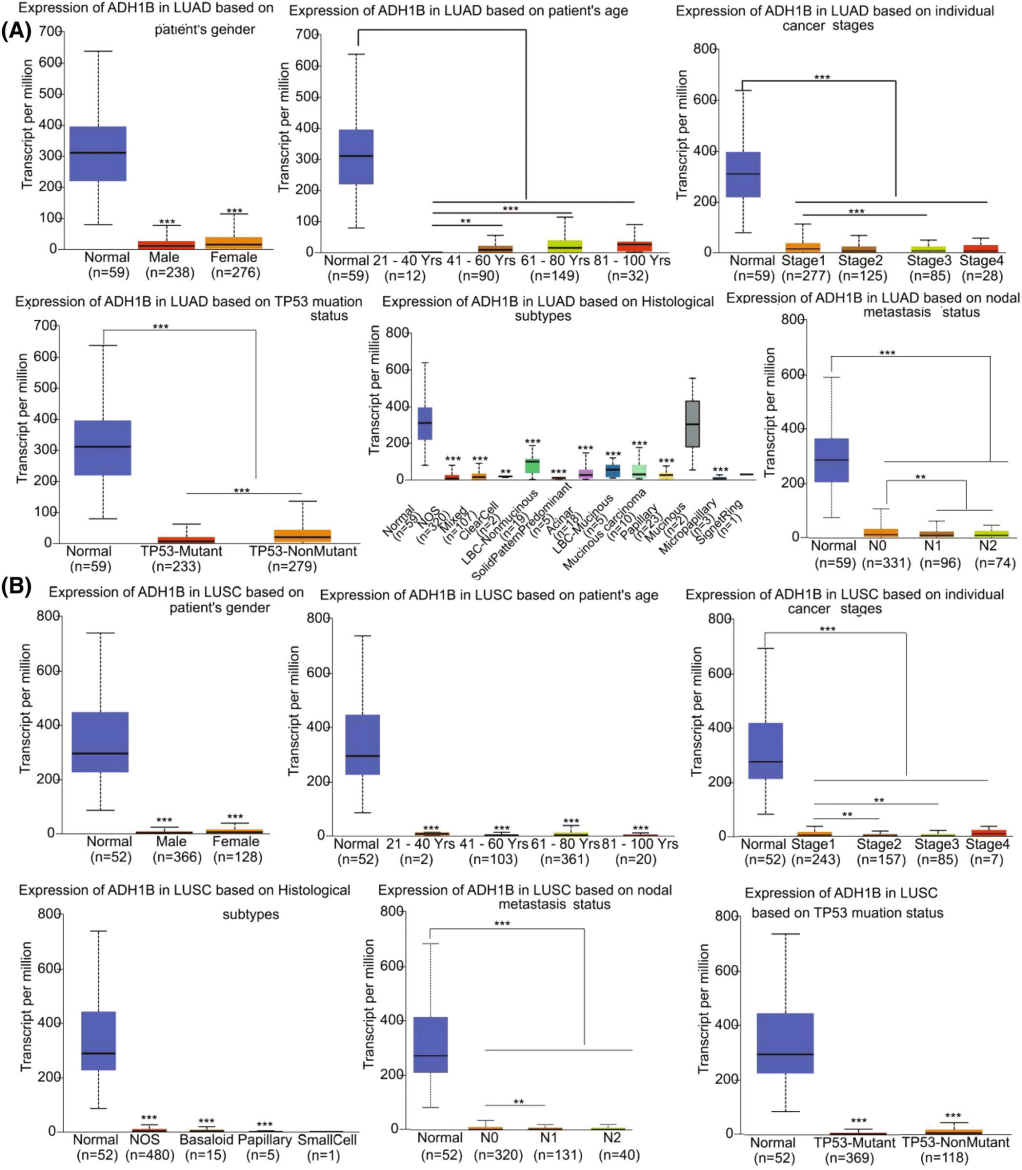
An analysis of the clinical factors affecting *ADH1B* expressions in LUAD and LUSC by the UALCAN database. (A) an analysis of the clinical factors affecting *ADH1B* expression in LUAD. (B) an analysis of the clinical factors affecting *ADH1B* expression in LUSC. ^**^ *P* < 0.01, ^***^ *P* < 0.001. *ADH1B*, alcohol dehydrogenase 1B; LBC‐mucinous, lung bronchioloalveolar carcinoma; LBC‐nonmucinous, lung bronchioloalveolar carcinoma non‐mucinous; LUAD, lung adenocarcinoma; LUSC, lung squamous cell carcinoma; Yrs, years; NOS, lung adenocarcinoma‐not otherwise specified; mixed, lung adenocarcinoma mixed subtype; N0, no regional lymph node metastasis; N1, metastases in 1 to 3 axillary lymph nodes; N2, metastases in 4 to 9 axillary lymph nodes.

We analyzed the correlation of *TOP2A* and *ADH1B* expression levels in LUAD and LUSC cases with several parameters such as patients' gender, age, individual cancer stage, histological subtype, nodal metastasis status, and TP53 mutation status, in the UALCAN database, respectively. As shown in Figure 3A, greater *TOP2A* expression levels were observed in male LUAD patients as compared to female patients (*P* < 0.05). Moreover, this enhanced *TOP2A* expression also increased with age, thus peaking at 41–60 years (*P* < 0.01). Additionally, *TOP2A* expression levels were higher in tumor tissues than in the normal tissues at each cancer stage (*P* < 0.01). Furthermore, *TOP2A* expression was positively correlated with histological subtypes, such as LBC‐Nonmucinous (Lung Bronchioloalveolar Carcinoma Non‐mucinous), solid predominant adenocarcinoma and adenocarcinoma with mixed subtypes. *TOP2A* expression levels were higher in LUAD and LUSC patients with 1–3 axillary lymph nodes metastases (N1) than in patients without any regional lymph node metastases (N0) or normal. Enhanced *TOP2A* expression levels were observed in TP53‐mutant LUAD patients more than in TP53‐nonmutant patients. Thus, *TOP2A* expressions in LUSC and LUAD cases were similar (Figure 3B). *ADH1B* expressions were similar in LUAD (Figure 4A) and LUSC (Figure 4B). As expected, they were opposite to *TOP2A* expressions in LUAD and LUSC cases.

### 3.4 Survival analysis of *TOP2A* and *ADH1B* levels in LUAD and LUSC

**FIGURE 5:**
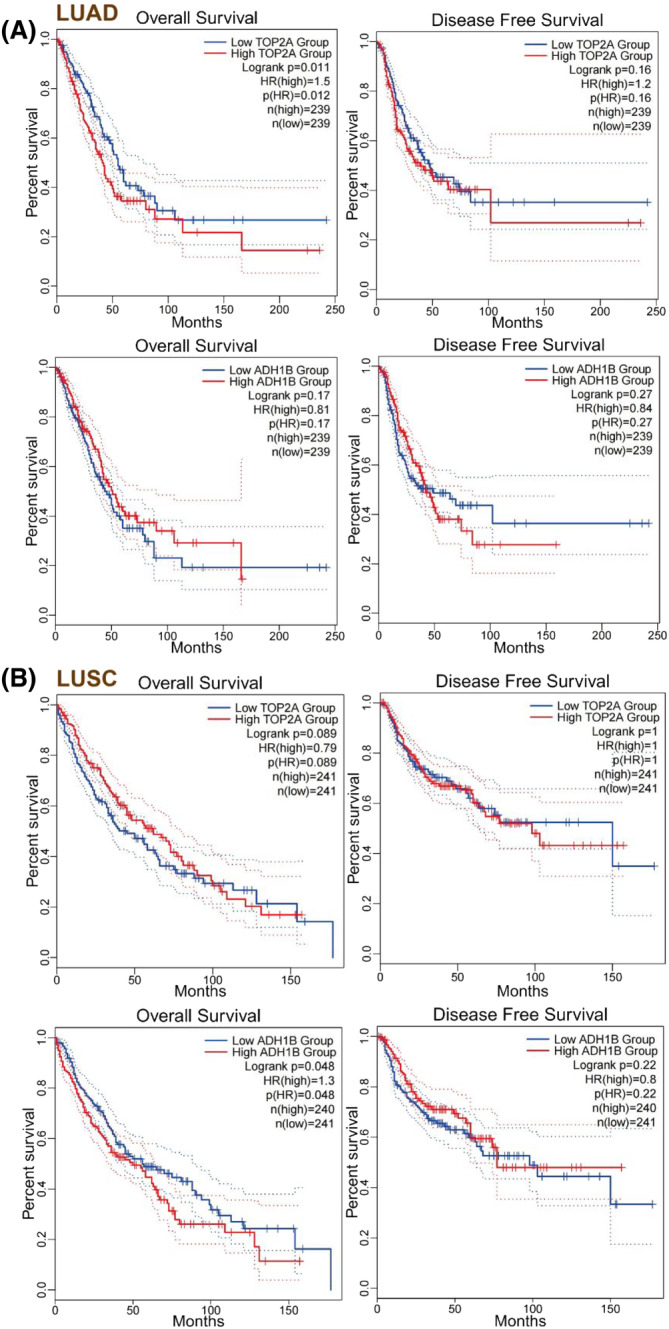
A survival analysis of TOP2A and ADH1B expressions in LUAD and LUSC by the GPEIA2 online tool. (A) Analysis of the effect of *TOP2A* and *ADH1B* expressions on the survival of LUAD patients. (B) Analysis of the effect of *TOP2A* and *ADH1B* expressions on the survival of LUSC patients. *ADH1B*, alcohol dehydrogenase 1B; LUAD, lung adenocarcinoma; LUSC, lung squamous cell carcinoma; *TOP2A*, topoisomerase II alpha.

**FIGURE 6:**
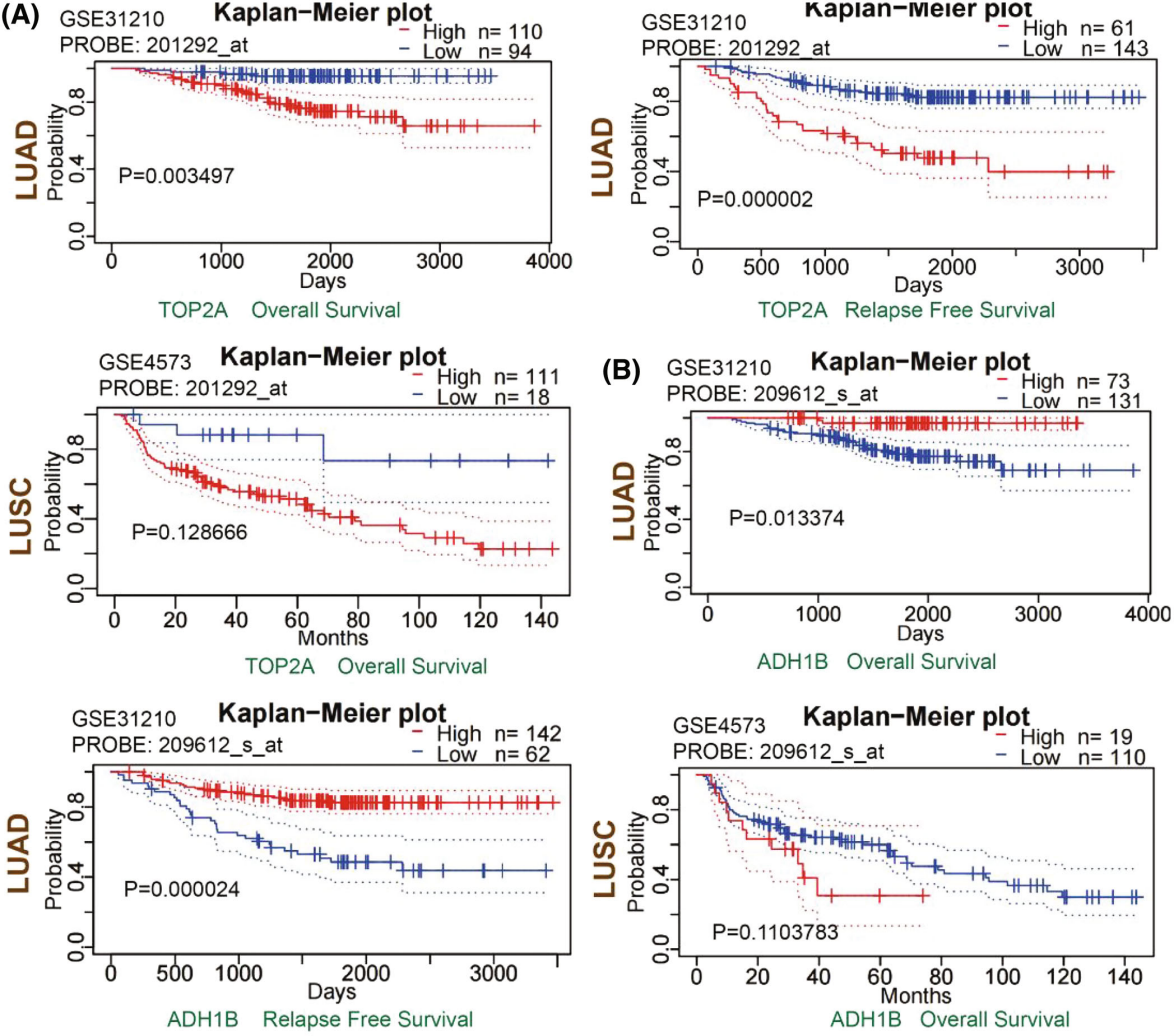
A survival analysis of TOP2A and ADH1B expressions in LUAD and LUSC by the PrognoScan online tool. (A) Analysis of the effect of *TOP2A* expressions on the survival of LUAD and LUSC patients. (B) Analysis of the effect of *ADH1B* expressions on the survival of LUAD and LUSC patients. *ADH1B*, alcohol dehydrogenase 1B; LUAD, lung adenocarcinoma; LUSC, lung squamous cell carcinoma; *TOP2A*, topoisomerase II alpha.

We analyzed the effects of *TOP2A* and *ADH1B* expressions on the survival of LUAD and LUSC patients, respectively. In the GPEIA2 database, as shown in Figure 5A, LUAD patients having higher *TOP2A* expressions had lower OS when compared with LUAD patients with low TOP2A expressions (*P* = 0.012), while DFS did not show any significant difference (*P* = 0.16). Additionally, *ADH1B* exhibited no significant effects on OS (*P* = 0.17) and DFS (*P* = 0.27) in LUAD patients. As seen in Figure 5B, *TOP2A* displayed no significant effects on OS (*P* = 0.089) and DFS (*P* = 1) in LUSC patients. However, LUSC patients with low *ADH1B* expression exhibited lower OS when compared with LUSC patients with higher *ADH1B* expressions (*P* = 0.048); DFS did not show any significant difference in such patients (*P* = 0.22). Furthermore, in the PrognoScan database, LUAD patients with higher *TOP2A* expressions exhibited lower OS and RFS than LUAD patients with low *TOP2A* expression (Figure 6A). On the contrary, LUAD patients with low *ADH1B* expression displayed lower OS and RFS than LUAD patients with higher *ADH1B* expression (Figure 6B). Thus, neither *TOP2A* (Figure 6A) nor *ADH1B* (Figure 6B) levels had a significant effect on the OS of LUSC patients.

### 3.5 Correlation analysis of TOP2A and ADH1B expressions with lipid metabolism regulatory genes in clinical practice

**FIGURE 7:**
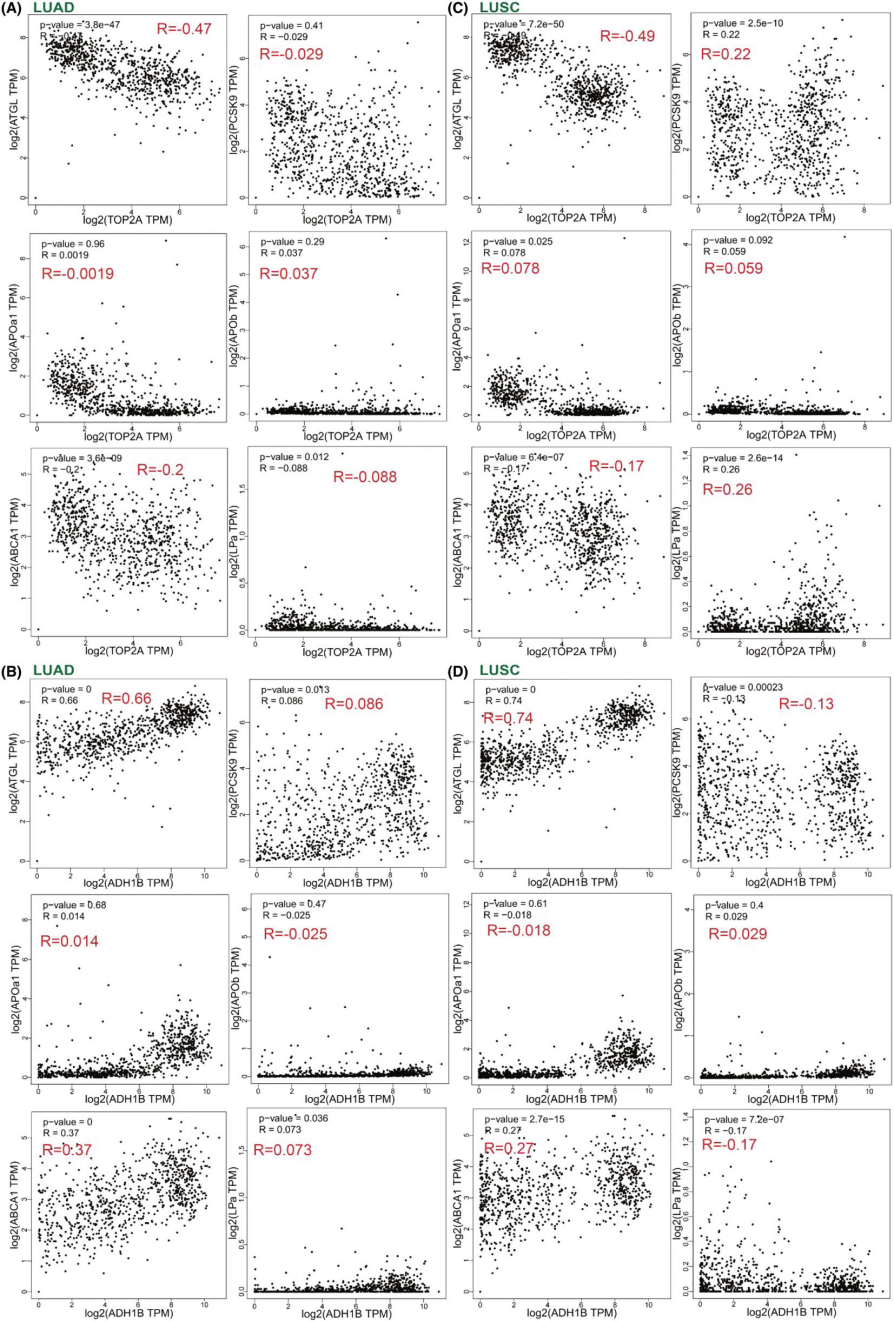
A correlation analysis of *TOP2A* and *ADH1B* expressions with lipid metabolism regulatory genes in clinical practice by the GPEIA2 online tool. (A) Correlation of *TOP2A* expression with genes regulating lipid metabolism in LUAD patients. (B) Correlation of *ADH1B* expression with genes regulating lipid metabolism in LUAD patients. (C) Correlation of *TOP2A* expression with genes regulating lipid metabolism in LUSC patients. (D) Correlation of *ADH1B* expression with genes regulating lipid metabolism in LUSC patients. *ATGL*, adipose triglyceride lipase; *PCSK9*, proprotein convertase subtilisin/kexin 9; *APOa1*, apolipoprotein a; *APOb*, apolipoprotein b; *ABCA1*, ATP‐binding cassette transporter A1; *TOP2A,* topoisomerase II alpha; *ADH1B*, alcohol dehydrogenase 1B; LUAD, lung adenocarcinoma; LUSC, lung squamous cell carcinoma.

As seen in Figure 7A,B, *TOP2A* displayed a negative correlation with *ATGL*, *ABCA1* and *LPa*, a driver gene of lipoprotein(a) levels, whereas *ADH1B* exhibited a positive correlation with *ATGL*, *ABCA1*, and *LPa* in LUAD cases. Additionally, *TOP2A* displayed a very weak negative correlation with *ATGL* and *ABCA1* and a very weak positive correlation with *PCSK9* and *LPa*, while *ADH1B* exhibited a very weak positive correlation with *ATGL* and *ABCA1* and a very weak negative correlation with *PCSK9* and *LPa* in LUSC patients (Figure 7C,D).

### 3.6 Clinical analysis of lipid levels in LUAD and LUSC patients

*TOP2A* upregulated and *ADH1B* down‐regulated expressions in LUAD and LUSC patients were associated with changes in lipid metabolism regulatory genes. Thus, we collected clinical data from 76 NSCLC and 61 non‐lung cancer patients for analyzing the lipid level changes in LUAD and LUSC patients in clinical settings. No significant differences were observed between the two groups in terms of gender (*P* = 0.379) and age (*P* = 0.105), whereas TC (*P* = 0.007) level was significantly higher in NSCLC patients as compared with non‐lung cancer patients, as seen in Table 2. Furthermore, multivariate logistic regression models showed that elevated TG (OR = 1.60; 95% CI = 1.02 to 2.49; *P* < 0.05) and TC (OR = 2.36; 95% CI = 1.04 to 5.34; *P* < 0.05) levels increased the risk of LUAD, and elevated TC (OR = 3.50; 95% CI = 1.33 to 9.19; *P* < 0.05) levels increased the risk of LUSC (Table 3). After adjusting for confounders (gender and age), the association of TC and TG levels with the risk of LUAD persisted, thereby indicating that elevated TG (OR = 1.59; 95% CI = 1.01 to 2.49; *P* < 0.05) and TC (OR = 2.45; 95% CI = 1.08 to 5.57; *P* < 0.05) levels may still increase the risk of LUAD (Table 3).

**TABLE 3:** Analyzing the association between blood lipid levels and NSCLC risk.

	Model before adjustment for confounders			Model after adjusting for confounding factors		
	Ref	LUSC OR 95% CI	LUAD OR 95% CI	Ref	LUSC OR 95% CI	LUAD OR 95% CI
TC	1	3.50 (1.33, 9.19)*	2.36 (1.04, 5.34)*	1	2.96 (0.97, 9.05)	2.45 (1.08, 5.57)*
TG	1	1.04 (0.60, 1.82)	1.60 (1.02, 2.50)*	1	0.80 (0.40, 1.60)	1.59 (1.01, 2.49)*
HDL	1	0.11 (0.02, 1.20)	0.85 (0.28, 2.64)	1	0.11 (0.01, 1.13)	0.80 (0.25, 2.60)
LDL	1	0.65 (0.34, 1.26)	1.17 (0.74, 1.85)	1	0.68 (0.30, 1.53)	1.16 (0.74, 1.84)
APOa	1	0.20 (0.02, 2.21)	0.87 (0.16, 4.85)	1	0.06 (0.01, 1.36)	0.82 (0.14, 4.82)
APOb	1	1.09 (0.07, 17.42)	5.78 (0.72, 46.98)	1	0.59 (0.02, 20.37)	5.63 (0.68, 46.30)
Lp(a)	1	1.00 (0.99, 1.01)	1.00 (0.99, 1.00)	1	1.00 (0.99, 1.00)	1.00 (0.99, 1.00)

Abbreviations: APOa, apolipoprotein a; APOb, apolipoprotein b; HDL, high‐density lipoprotein; LDL, low‐density lipoprotein; Lp(a), lipoprotein(a); LUAD, lung adenocarcinoma; LUSC, lung squamous cell carcinoma; NSCLC, non‐small cell lung cancer; TC, total cholesterol; TG, triglyceride.

*Note:* Adjusted for age and gender.

* *P* < 0.05.

## 4 DISCUSSION

Our study revealed that although *TOP2A* and *ADH1B* genes can be used as diagnostic markers for LUAD and LUSC, they can only be used as independent prognostic factors for LUAD patients. Furthermore, *TOP2A* and *ADH1B* expressions correlated with genes regulating lipid metabolism, while elevated TG and TC levels increased the risk of LUAD but not LUSC. Thus, *TOP2A* and *ADH1B* may influence the prognosis of LUAD patients by regulating their lipid metabolism.

Initial tumor development involves several disordered genetic expressions.^21^ Key prognostic biomarkers for diagnosing NSCLC have been identified by bioinformatic analysis,^22^ but subtype integration analysis has not been performed to date. Although LUAD and LUSC display varied pathological and clinical features, they might share common driver genes. We identified the expressions of *TOP2A*, a common upregulated gene, and *ADH1B*, a common downregulated gene, in LUAD and LUSC cases through multiple gene datasets. Although the *TOP2A* and *ADH1B* expressions in both LUAD and LUSC correlated with the patient's gender, age, individual cancer stage, histological subtype, nodal metastasis status, and TP53 mutation status, they demonstrated different effects on LUAD and LUSC patients' survival. LUAD patients with high *TOP2A* and low *ADH1B* expressions displayed poor survival, while *TOP2A* and *ADH1B* expressions had no significant effect on the survival of LUSC patients. Thus, these results suggest that *TOP2A* and *ADH1B* can be used as diagnostic markers for LUAD and LUSC but only as independent prognostic factors for LUAD and not for LUSC.

Abnormal lipid metabolism plays an important role in promoting tumorigenesis and progression by inducing impaired antitumor‐immune responses.^23^, ^24^ Hence, abnormal lipid metabolism accompanying tumorigenesis and progression can lead to abnormal lipoprotein levels in patients with malignant tumors. A study has shown that elevated serum lipid levels can be an early sign of metastasis in NSCLC patients.^3^ In clinical practice, our main indicators for monitoring lipid levels include TC, TG, high‐density lipoprotein (HDL), low‐density lipoprotein (LDL), APOa, APOb, and lipoprotein(a) [Lp(a)] levels. Thus, we combined these common clinical lipid indicators with a thorough literature review to identify *ATGL*, *PCSK9*, *ABCA1*, *APOa1*, *APOb*, and *LPa* as the lipid metabolism regulatory genes to be analyzed in our study. ATGL is a rate‐limiting enzyme that mobilizes fatty acids from cellular triglyceride stores and also mainly contributes to TG catabolism. Hence, the resultant fatty acid metabolism dysregulation is closely associated with dyslipidemia and metabolic disorders.^25^ PCSK9 is a key protein in LDL metabolism and helps in the degradation of LDL receptors.^26^ ABCA1 is an important protein for maintaining cholesterol homeostasis, promoting intracellular excess cholesterol and phospholipid efflux, and controlling the rate‐limiting step of the reverse cholesterol transport mechanism.^27^ APOa1 is the major plasma HDL lipoprotein that exhibits several proven cardioprotective functions.^28^ APOb is a large amphiphilic glycoprotein crucial for human lipoprotein metabolism and is characterized by the induction of hypercholesterolemia and premature coronary artery disease.^29^ Additionally, the protein encoded by the *LPa* gene forms an important part of Lp(a), and Lp(a) is significantly associated with the progression and staging of lung cancer.^30^

Since lipid specimens are easy to collect, lipid levels are detected in a short time and closely followed in clinical work. The research on the association of serum lipid levels and the occurrence and prognosis of lung cancer, as a direct representative of lipid levels in the body, has also gained attention in recent years. Lipids, as modifiable risk factors, are of great importance in preventing lung cancer. Therefore, we first analyzed the correlation of *TOP2A* and *ADH1B* with the above‐mentioned lipid metabolism genes by the GEPIA2 online tool. Subsequently, we found that *TOP2A* and *ADH1B* expressions were closely associated with *ATGL*, *ABCA1*, *PCSK9*, and *LPa* gene expression levels in LUAD and LUSC cases. Furthermore, after collecting lipid panel data from clinical patients, we found that although both TC and TG levels were elevated in NSCLC patients when compared to non‐lung cancer patients, the elevated TG and TC levels only increased the risk of LUAD and not LUSC. Hence, this might be the reason for the low survival rate of LUAD patients due to abnormal *TOP2A* and *ADH1B* expressions. However, further studies are needed to determine whether *TOP2A* and *ADH1B* expressions can regulate lipid metabolism gene levels in LUSC patients that affect patients' prognosis.

However, our study has some limitations. First, we did not validate *TOP2A* and *ADH1B* expressions and their relationship with lipid metabolism at in vivo and in vitro levels. Second, a larger sample size is needed to analyze and validate the relationship between lipid levels and NSCLC typing and staging. Thus, we will further investigate the molecular mechanisms of *TOP2A* and *ADH1B* genes in lipid metabolism regulation and lung cancer progression in upcoming studies.

## 5 CONCLUSIONS

Our study shows that *TOP2A* and *ADH1B* genes can be used as diagnostic markers for LUAD and LUSC, but only as independent prognostic factors and lipid metabolism regulatory genes for LUAD patients and not LUSC cases. This combination of *TOP2A* and *ADH1B* gene diagnostics and clinical lipid panel investigations might be an effective intervention for early and accurate diagnosis, targeted therapy, and improved prognosis of LUAD, thus enhancing the current clinical management strategies of lung cancer.

## AUTHOR CONTRIBUTIONS

*Conception and design*: D Yin, Y Zhang, and L Cheng. *Administrative support*: L Cheng. *Provision of study materials or patients*: D Yin and H Li. *Collection and assembly of data*: D Yin and H Li. *Data analysis and interpretation*: Y Zhang. *Manuscript writing*: All authors. *Final approval of manuscript*: All authors.

## CONFLICT OF INTEREST STATEMENT

The authors have declared that no competing interest exists.

## ETHICS STATEMENT

The authors are accountable for all aspects of the work in ensuring that questions related to the accuracy or integrity of any part of the work are appropriately investigated and resolved. The study was conducted in accordance with the Declaration of Helsinki (as revised in 2013). The study was approved by the Human Research Ethics Committee of the First Affiliated Hospital (Huainan First People's Hospital) of Anhui University of Science and Technology, China (No. EC‐20221028‐1013), and individual consent for this retrospective analysis was waived.

## Data Availability

The data that support the findings of this study are available on request from the corresponding author. The data are not publicly available due to privacy or ethical restrictions.
